# Unsafe abortion and abortion-related death among 1.8 million women in India

**DOI:** 10.1136/bmjgh-2019-001491

**Published:** 2019-05-02

**Authors:** Ryo Yokoe, Rachel Rowe, Saswati Sanyal Choudhury, Anjali Rani, Farzana Zahir, Manisha Nair

**Affiliations:** 1 Nuffield Department of Population Health, University of Oxford, Oxford, UK; 2 NPEU, Nuffield Department of Population Health, University of Oxford, Oxford, UK; 3 Department of Obstetrics and Gynaecology, Guwahati Medical College and Hospital, Guwahati, India; 4 Department of Obstetrics and Gynaecology, Institute of Medical Sciences, Banaras Hindu University, Varanasi, India; 5 Department of Obstetrics and Gynaecology, Assam Medical College, Dibrugarh, India

**Keywords:** abortion, unsafe, rates, risk factors, India

## Abstract

**Introduction:**

Unsafe abortion is a preventable cause of maternal mortality. While studies report high number of abortions in India, the population-level rates of unsafe abortion and their risk factors are not well understood. Our objective was to analyse the rates of and risk factors for unsafe abortion and abortion-related maternal death in India.

**Methods:**

We conducted a secondary analysis of data from 1 876 462 pregnant women aged 15–58 years from nine states in the Indian Annual Health Survey (2010–2013). We calculated the rate of unsafe abortion and abortion-related mortality with 95% CI. Multivariable logistic regression models examined the associations of sociodemographic characteristics, health seeking behaviours and family planning with unsafe abortion and abortion-related mortality.

**Results:**

There were 89 447 abortions among 1 876 462 pregnant women in 2007–2011 (4.8%; 95% CI 4.8 to 4.9). Of these, 58 266 were classified as unsafe (67.1%; 95% CI 66.7 to 67.5). There were 253 abortion-related maternal deaths (0.3%; 95% CI 0.2 to 0.3). Factors associated with unsafe abortion: maternal age 20–24 years (adjusted OR (aOR): 1.13; 95% CI 1.09 to 1.18), illiteracy (aOR: 1.48; 95% CI 1.39 to 1.59), rural residence (aOR: 1.26; 95% CI 1.21 to 1.32), Muslim religion (aOR: 1.16; 95% CI 1.12 to 1.22), Schedule caste social group (aOR: 1.08; 95% CI 1.04 to 1.12), poorest asset quintile (aOR: 1.45; 95% CI 1.38 to 1.53), antenatal care (aOR: 0.69; 95% CI 0.67 to 0.72), no surviving children (aOR: 1.30; 95% CI 1.16 to 1.46), all surviving children being female (aOR: 1.12; 95% CI 1.07 to 1.17), use of family planning methods (aOR: 0.69; 95% CI 0.66 to 0.71). Factors associated with abortion-related deaths: maternal age 15–19 (aOR: 7.79; 95% CI 2.73 to 22.23), rural residence (aOR: 3.28; 95% CI 1.76 to 6.11), Schedule tribe social group (aOR: 4.06; 95% CI 1.39 to 11.87).

**Conclusion:**

Despite abortion being legal, the high estimated prevalence of unsafe abortion demonstrates a major public health problem in India. Socioeconomic vulnerability and inadequate access to healthcare services combine to leave large numbers of women at risk of unsafe abortion and abortion-related death.

**Key questions**

**What is already known?**

- There is a high prevalence of unsafe abortion in India, but population level rates and risk factors are not clearly understood.

**What are the new findings?**

- 67% of abortions in the study population in India were classified as unsafe, varying widely across the states (range 45.1%–78.3%).
- There was a disproportionately higher risk of unsafe abortion among the vulnerable and disadvantaged populations in India.
- Young women aged 15–19 years were at the highest risk of dying from an abortion-related complication.

**What do the new findings imply?**

- Urgent work is needed to understand the barriers to safe abortion in India, despite the conducive legal environment.

## Introduction

Unsafe abortion is one of the preventable causes of maternal mortality^1^ yet, of the 55.7 million abortions that occurred globally each year between 2010 and 2014, an estimated 25.1 million (45.1%) were unsafe.^2^ Defined by the WHO as “the termination of an unintended pregnancy either by persons lacking the necessary skills or in an environment lacking the minimum medical standards or both,”^3^ unsafe abortion is strongly associated with maternal complications such as haemorrhage, sepsis and trauma, and is the fourth leading cause of maternal death.^4^ Abortion plays a crucial role in the reproductive health of Indian women.^5^ An estimated 15·6 million abortions (14.1 million–17.3 million) were conducted in India in 2015. Women in India often turn to unqualified providers for abortion,^6^ despite abortion being made legal in the country through the Medical Termination of Pregnancy Act in the early 1970s.^7^ While several studies suggest a high prevalence of unsafe abortion and related complications among women of reproductive age group in India,^2 6^ ^8 9^ population-level rates of unsafe abortion and abortion-related mortality, and their risk factors are not well understood.

Previous research and theoretical arguments on abortion in India point to three main and interrelated factors that are important in understanding the context of seeking abortion: (1) women’s labour force participation and educational attainment; (2) women’s social class and ethnicity; (3) the predominant preference for male children.^10^ However, the combined effect of these factors has not been tested empirically. This is crucial to identify populations that are at a higher risk of seeking unsafe abortion in India to prevent maternal complications and deaths. The objectives of this study were to: (1) estimate the rates of unsafe abortion and abortion-related maternal mortality in nine states in India; (2) examine the sociodemographic characteristics of women who have an abortion compared with women who have a live birth; (3) investigate the risk factors for unsafe abortion; (4) investigate the risk factors for abortion-related maternal death in India.

## Methods

We conducted a secondary data analysis of the 2010–2013 round of India’s Annual Health Survey (AHS) to analyse the rate of and risk factors for unsafe abortion and abortion-related maternal deaths in nine states in India.

### Definitions

Based on the WHO definition, we used three criteria to identify ‘unsafe abortions’ using AHS data: (1) the setting where the abortion was performed (if induced) or completed (if spontaneous); (2) the person who performed or completed the abortion; (3) the gestational age at which the abortion was performed or completed. Abortions were classified as unsafe if they were not performed or completed in a health facility, not performed or completed by a skilled birth attendant, or performed or completed at 20 weeks of gestation (~5 months) or beyond. Abortions at or beyond 20 weeks’ gestation were classified as unsafe because of the association with increased risk of maternal morbidity and mortality^11^ and because abortion beyond 20 weeks of pregnancy is illegal in India and under such a condition woman may be forced to seek abortion services from unqualified providers. The breakdown of unsafe abortion according to the three criteria is presented in online supplementary table S1.

10.1136/bmjgh-2019-001491.supp1 Supplementary data

We combined induced and spontaneous abortion into one category to minimise the risk of misclassification^3 10^as most induced abortions are unreported or reported as spontaneous in surveys for legal, ethical and moral reasons.^12 13^Further, it was considered that determining safety of abortion was more important than examining types of abortion. Rees *et al* have argued that both induced and spontaneous abortion can result in unsafe abortion and present with complications.^14^

### Data source

We used AHS (2010–2013) data. The AHS is a population-based household survey in which self-reported data on maternal and child health, demographics, birth and access to health and family planning services were collected from 4.3 million households in nine less developed states of India (Bihar, Chhattisgarh, Jharkhand, Madhya Pradesh, Odisha, Rajasthan, Uttar Pradesh, Uttarakhand and Assam), representing 50% of the country’s population, 61% of births and 62% of maternal deaths.^15 16^The AHS used a stratified simple random sampling (without replacement) to obtain a sample that was representative of and proportional to the size of the selected villages. Survey weights were developed to account for the sampling method. The survey administered four ‘schedules’ (or questionnaires): (1) House-listing Schedule, (2) Household Schedule, (3) Women Schedule and (4) Mortality Schedule. Relevant data from all four schedules were merged for this study. Detailed objectives and associated methodology can be found in the AHS Report (Part I. 2014).^16^

### Study sample

All women who provided information on their pregnancy (91.3% most recent pregnancy and 8.7% on a previous pregnancy) were included. As in a previous study using the same dataset, women who had an abortion after 28 weeks were excluded as these were most likely to be stillbirths (according to the WHO definition for stillbirth).^15^ A total of 1 876 462 women who reported being pregnant during the reference period 2007–2011 were included in the study. The mortality data were extracted from the mortality Schedule of the AHS and 253 abortion-related deaths were included, giving a total of 89 447 abortions in 2007–2011. Among these, 253 women who died and 83 women who survived did not have information to examine the safety of abortion. Therefore, safety of abortion was examined in a total of 89 111 women, of which 58 266 had unsafe abortions and 30 845 had safe abortions. online supplementary figure S1 further illustrates how we derived the samples for each study objective.

### Potential risk factors for unsafe abortion and abortion-related deaths

We conducted a systematic search and review of the literature to identify risk factors for unsafe abortion and abortion-related death. Informed by the literature review, we developed conceptual frameworks to map the relationships of the risk factors with unsafe abortion (online supplementary file 1) and abortion-related mortality (online supplementary file 1) according to proximity to the outcome, and to guide selection of variables and analysis. Based on the literature review and conceptual frameworks, we grouped the population characteristics/potential risk factors as sociodemographic characteristics, pregnancy-related characteristics, family characteristics, the use of family planning methods, and health seeking behaviours and mapped these against the available data in the AHS. We used survey data about household assets and principle component analysis^17^ to derive a measure of household wealth which is thought to be a good proxy of economic status.^18 19^We used data about the number of surviving children and the number of female children to derive the proportion of surviving female children. We considered the following as potential risk factors for unsafe abortion and/or abortion-related death: use of family planning method; place of residence; social group; religion; asset index/wealth; number of total surviving children; proportion of female children; maternal age; maternal education status; antenatal care (ANC) use; marital status; maternal employment; gestational month of abortion. The variables and their categorisation are described in online supplementary file 1. All independent variables reflect characteristics of the household or women at the point of the survey. Baseline groups were chosen as the group with the least potential risk of having unsafe abortion, except for the use of family planning methods and the use of ANC (baseline—higher potential risk).

### Statistical analysis

There were three outcomes of interest: (1) the outcome of the woman’s pregnancy (live birth or abortion); (2) the safety of abortion (safe or unsafe); (3) the outcome of abortion (survived or died). The rate of abortion, unsafe abortion, and abortion-related death and the corresponding 95% CIs were calculated. The denominator for abortion rate was the total number of pregnancies in the reference period (2007–2011), and for the rates of unsafe abortion and abortion-related death was the total number of abortions during the same reference period. The characteristics of women who had an abortion were compared with those who had a live birth. We used univariable logistic regression analysis to examine the association between each independent variable and the outcomes (unsafe abortion and abortion-related mortality). Modelling a non-linear association between maternal age and the outcomes using fractional polynomials showed that maternal age acted in a non-linear fashion and was therefore used as a categorical variable.

Multivariable models were built using a stepwise forward regression approach, with our conceptual frameworks used to select the order for including the variables starting from distal to proximal (online supplementary figures S2 and S3). During model building for unsafe abortion we used a p value <0.05 in the univariable analysis as a cut-off for including a variable. We used the Wald test at the 5% significance level to determine if adding a variable significantly improved the model fit. In the multivariable model examining the risk factors for abortion-related death we chose to include all six potential risk factors, regardless of the results of the univariable analysis, because the number of variables available in the mortality dataset was small, and in order to control for confounding effects. Factors whose effects were attenuated by other variables in the multivariable regression were further examined to identify confounding. We calculated the proportion of factors reported to contribute to abortion-related maternal death.

Collinearity between independent variables was explored using pairwise correlation coefficients. We tested for interactions between variables for which there was a strong theoretical rationale. In the risk factor analysis for unsafe abortion, we therefore tested for interactions between employment and residence, employment and wealth, and social group and wealth. In the risk factor analysis for abortion-related mortality, interactions between social group and wealth, and social group and residence were examined. Potential interactions observed using univariable logistic regression were further assessed using the Wald test comparing the multivariable model with the relevant interaction terms with an empty model. No significant interactions were found at the 5% significance level.

We carried out an exploratory post hoc subgroup analysis to investigate the effect of the number of surviving female children in households where all children were female on the odds of unsafe abortion. All statistical analyses were carried out using Stata V.13.1 using the ‘*svy set’* function to account for the stratified and clustered nature of the data. All proportions, means and CIs presented are therefore weighted for design effects and non-response. Two-sided p values <0.05 were taken to indicate statistical significance.

Missing observations per variable were quantified, and we explored the ‘type of missingness’ by generating a new variable indicating missing data for each risk factor followed by logistic regression analysis to identify factors that predicted missingness. Based on this analysis, data were assumed to be ‘missing at random’ and three methods were used to address bias due to missing data: missing indicator method, complete case analysis and multiple imputation.^20^ The ‘missing indicator’ model in which missing data were grouped as a separate category was used as the final model. However, to maintain model stability, for variables that had <1% missing data, a separate category for ‘missing’ was not generated.

### Study power

For the fixed sample size of 89 111 women who were classified as having a safe abortion and 58 266 who had an unsafe abortion, this study had 90% power to detect an OR of ≥1.29 or ≤0.75 associated with unsafe abortion at p<0.05 (two-tailed) for the risk factor with the lowest prevalence (‘other religion’ 0.6%), and an OR of ≥1.43 or ≤0.74 for the risk factor with the highest prevalence (‘being married’ 99.6%) in the study population.

For the fixed sample size of 89 447 women who had an abortion and survived and 253 women who died during or within 42 days of the abortion procedure, this study had 80% power to detect an OR of ≥3.00 associated with abortion-related death at p<0.05 (two-tailed) for the risk factor with the lowest prevalence (‘Christian religious group’ 0.6%), but not enough power to detect an OR less than one at a clinically meaningful level. This study had 80% power to detect an OR of ≥1.43 or ≤0.74 for the risk factor with the highest prevalence (gestational month of abortion <5; 99.6%) in the study population.

### Patient and public involvement

This is not applicable since this was a secondary analysis of anonymous survey data.

## Results

### Rate of abortion, unsafe abortion and abortion-related mortality

**Figure 1:**
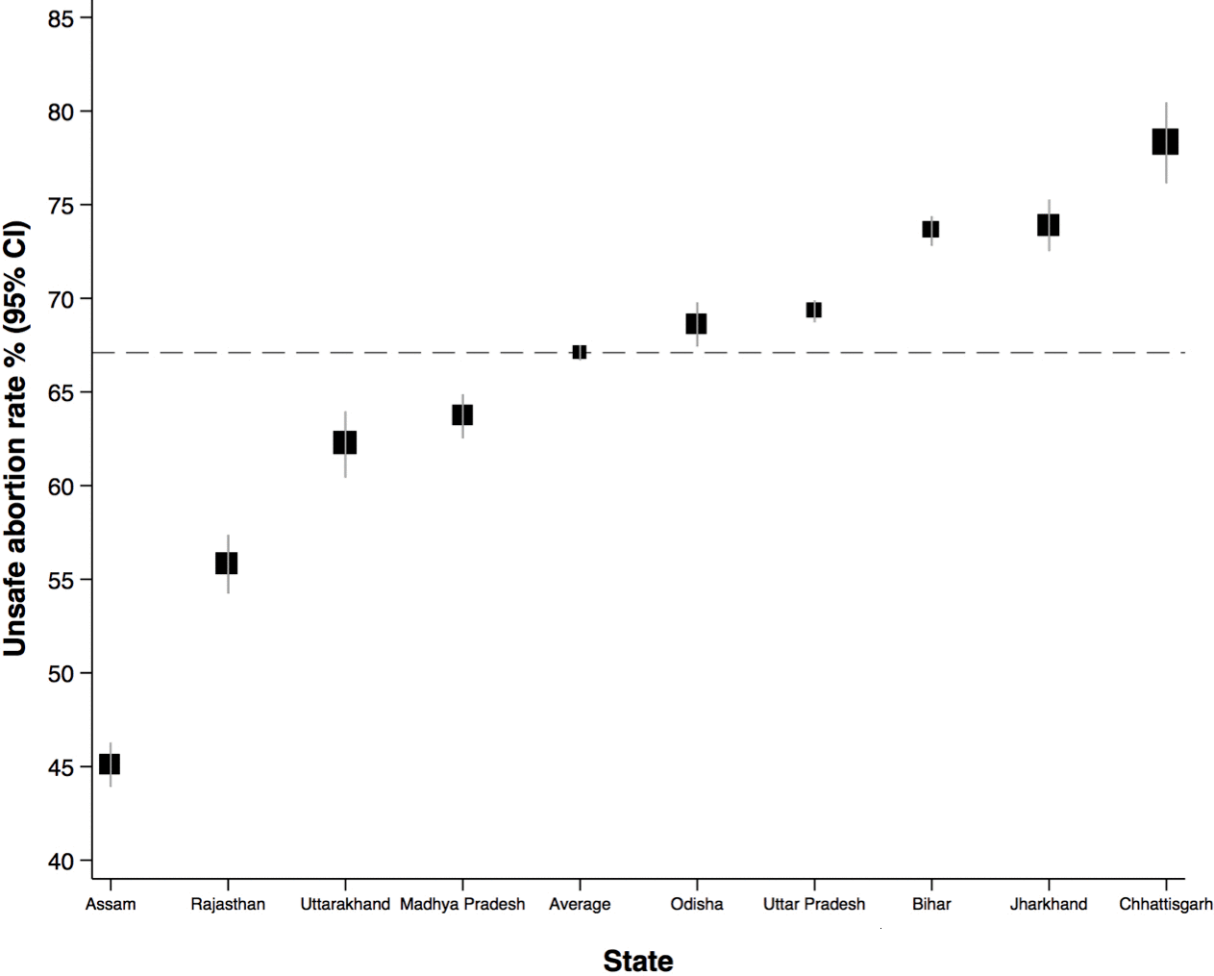
Rate of unsafe abortion in nine states in India.

Among a total of 1 876 462 pregnant women in the study population, 89 194 women had an abortion leading to an overall rate of 4.8% (95% CI 4.8 to 4.9). The rate of abortion for each state is shown in table 1. The prevalence of abortion was highest in Assam (6.5%) and the lowest in Chhattisgarh (1.6%). Out of 89 111 women who survived and had sufficient information to examine the safety of abortion, 58 266 women were classified as having an unsafe abortion. The overall rate of unsafe abortion was 67.1% (95% CI 66.7 to 67.5) with five out of nine states above the overall rate (online supplementary table S3, figure 1). There was a large variation in the rate across the states: Assam had the lowest (45.1%) and Chhattisgarh has the highest rate of unsafe abortion (78.3%). Among a total of 89 194 women who had an abortion, 253 were reported as abortion-related maternal death in the AHS, giving an abortion-related mortality rate of 0.3% (95% CI 0.2 to 0.3) (online supplementary table S4).

**Table 1:** Number and rate of abortion in nine states in India, using the AHS 2012–2013

	Women who reported a pregnancy, who were alive at the time of the AHS, and did not have an abortion after28weeks’gestation, including those who died after having an abortion†	Women who had an abortion, including those who died after having an abortion†	Abortion rate* (95%CI)
**Overall**	1 876 715	89 447	4.8 (4.8 to 4.9)
Assam	165 842	12 306	6.5 (6.3 to 6.6)
Bihar	348 569	14 562	4.3 (4.2 to 4.3)
Chhattisgarh	105 774	1658	1.6 (1.5 to 1.7)
Jharkhand	149 140	6212	4.1 (4.0 to 4.3)
Madhya Pradesh	218 817	7041	3.3 (3.2 to 3.4)
Odisha	114 053	6984	5.8 (5.7 to 6.0)
Rajasthan	132 680	4897	3.5 (3.3 to 3.6)
Uttarakhand	145 923	6470	5.4 (5.2 to 5.5)
Uttar Pradesh	495 917	29 317	5.9 (5.8 to 6.0)

*The abortion rate is the number of abortions per 100 pregnancy outcomes (either an abortion or a live birth).

†Frequencies are unweighted (true counts). Rates/proportions are weighted for survey design and clustering effects.

AHS, Annual Health Survey.

The characteristics of women who had an abortion and those who had a live birth are presented in table 2. Overall, despite statistically significant differences because of the large sample size, there were no clinically meaningful differences in age, education, residence, religion or social class background between the two groups. Two factors showed wide variation between the abortion and live birth groups. While 85.1% of women who had a live birth reported having some ANC, only 23.1% of women who had an abortion had ANC. This was true even after excluding women who had an early abortion (<12 weeks). Also, a marginally higher proportion of women who had an abortion belonged to the highest quintiles of the asset index.

**Table 2:** Characteristics of the study population by pregnancy outcome

Characteristics	Women who had an abortion and survived* (n=89 194)	Women who had live births* (n=1 787 268)	P value†
Maternal age (years)			
15–19	3591 (4.0)	55 882 (3.0)	<0.001
20–24	25 775 (28.9)	591 640 (32.2)	
25–29	29 310 (32.3)	634 182 (35.2)	
30–34	17 640 (19.8)	310 726 (18.0)	
35–39	8975 (10.2)	132 393 (7.9)	
40–44	2903 (3.5)	44 215 (2.7)	
≥45	1000 (1.3)	18 214 (1.1)	
Missing	0 (0.0)	16 (0.0)	
Marital status			
Married	88 824 (99.6)	1 772 493 (99.2)	<0.001
Single	370 (0.4)	14 765 (0.8)	
Missing	0 (0)	10 (0)	
Maternal education			
Tertiary and above	5221 (5.8)	85 608 (4.5)	<0.001
Secondary school	29 703 (29.5)	495 845 (24.7)	
Primary school/below	22 053 (24.0)	433 415 (23.4)	
Illiterate	32 074 (40.6)	771 004 (47.2)	
Missing	143 (0.2)	1396 (0.1)	
Maternal employment status			
In-paid employment	11 130 (11.0)	269 348 (13.9)	<0.001
Not in-paid employment	77 471 (89.0)	1 505 539 (85.5)	
Missing	593 (0.6)	12 381 (0.7)	
Place of residence			
Urban	16 601 (21.4)	257 704 (16.8)	<0.001
Rural	72 593 (78.6)	1 529 564 (83.2)	
Missing	0 (0)	0 (0)	
Religion			
Hindu	73 340 (81.0)	1 468 352 (81.2)	<0.001
Muslim	13 541 (17.3)	267 750 (16.6)	
Christian	878 (0.8)	24 558 (1.2)	
Others	1293 (0.8)	25 298 (0.8)	
Missing	142 (0.2)	1310 (0.1)	
Social group			
Others	64 591 (73.2)	1 217 533 (68.8)	<0.001
Schedule caste	16 656 (19.9)	345 084 (20.5)	
Schedule tribe	7805 (6.7)	223 345 (10.6)	
Missing	142 (0.2)	1306 (0.1)	
Wealth/asset index			
5: Highest	20 678 (23.3)	329 699 (18.7)	<0.001
4	18 242 (20.4)	332 053 (18.7)	
3	16 452 (19.1)	328 566 (19.1)	
2	14 646 (15.9)	339 755 (18.2)	
1: Lowest	12 799 (14.1)	339 366 (18.7)	
Missing	6377 (7.1)	117 829 (6.6)	
Antenatal care use			
No	69 113 (76.9)	244 447 (14.9)	<0.001
Yes	20 081 (23.1)	1 542 821 (85.1)	
Missing	0 (0)	0 (0)	
Self-reported mental illness			
No	88,42 (99.1)	1 773 403 (99.2)	<0.001
Yes	627 (0.8)	12 505 (0.7)	
Missing	143 (0.2)	1360 (0.1)	
Number of surviving children at the point of interview			
0	2910 (3.3)	21 854 (1.3)	<0.001
1–3	55 971 (60.2)	1 366 546 (74.5)	
4 or more	13,92 (17.6)	394 976 (23.9)	
Missing	16 388 (19.0)	3892 (0.3)	
Proportion of surviving female children among total surviving children			
0%	19 825 (21.3)	464 452 (25.2)	<0.001
10%–30%	9710 (11.9)	250 552 (15.0)	
40%–60%	18 198 (20.6)	452 104 (25.8)	
70%–90%	7495 (8.6)	230 421 (13.1)	
100%	15 737 (16.7)	377 224 (20.0)	
Missing	18 229 (21.0)	12 515 (0.9)	
Family planning (at the point of interview)			
No	35 117 (39.7)	690 246 (38.7)	<0.001
Yes	38 960 (42.5)	832 645 (46.1)	
Missing	15 117 (17.8)	264 377 (15.2)	

*Frequencies are unweighted (true counts). Rates/proportions are weighted for survey design and clustering effects.

†P value shows two-sided p value for χ^2^ test for difference in proportions.

### Risk factors for unsafe abortion in India

The characteristics of the women who had an unsafe abortion and those who had a safe abortion are described in table 3. All sociodemographic characteristics (except marital status and maternal employment status) and all other characteristics (except self-reported mental illness) were found to be statistically significantly associated with unsafe abortion, and these associations were not substantially altered after adjustment for all potential risk factors (table 3).

**Table 3:** Unadjusted and adjusted associations between sociodemographic and family characteristics, health seeking behaviour, family planning and unsafe abortion

	Women who had unsafe abortion* (n=58 266)	Women who had safe abortion* (n=30 845)	Unadjusted OR (95% CI)† missing indicator (n=89 111)	P value	Adjusted OR‡ (95% CI)† missing indicator (n=89 111)	P value§
Sociodemographic characteristics						
Maternal age (years)						
15–19	2548 (4.2)	1041 (3.4)	1.22 (1.11 to 1.33)	<0.001	1.09 (1.00 to 1.18)	<0.001
20–24	17 852 (30.3)	7889 (24.6)	1.17 (1.12 to 1.23)		1.13 (1.09 to 1.18)	
25–29	18 932 (32.3)	10 354 (32.4)	1.00 (ref)		1.00 (ref)	
30–34	10 908 (18.9)	6716 (22.4)	0.86 (0.82 to 0.90)		0.88 (0.84 to 0.92)	
35–39	5508 (9.7)	3460 (12.0)	0.87 (0.82 to 0.92)		0.82 (0.78 to 0.87)	
40–44	1833 (3.4)	1070 (3.9)	0.94 (0.85 to 1.04)		0.82 (0.75 to 0.89)	
≥45	685 (1.3)	315 (1.3)	1.00 (0.86 to 1.18)		0.95 (0.83 to 1.09)	
Missing	0 (0)	0 (0)	N/A		N/A	
Marital status						
Married	58.012 (99.6)	30 729 (99.6)	0.91 (0.69 to 1.20)	0.5125	N/A¶	
Single	254 (0.4)	116 (0.4)	1.00 (ref)		N/A¶	
Missing	0 (0)	0 (0)	N/A		N/A¶	
Maternal education						
Tertiary and above	2970 (4.8)	2242 (7.7)	1.00 (ref)	<0.001	1.00 (ref)	<0.001
Secondary school	17 641 (27.3)	12 032 (34.2)	1.27 (1.18 to 1.37)		0.94 (0.88 to 1.00)	
Primary school/below	14 395 (23.8)	7644 (24.3)	1.56 (1.44 to 1.68)		1.13 (1.05 to 1.20)	
Illiterate	23 165 (43.9)	8879 (32.6)	2.07 (1.92 to 2.23)		1.48 (1.39 to 1.59)	
Missing	95 (0.2)	48 (0.1)	1.92 (1.21 to 3.05)		N/A**	
Maternal employment status						
In-paid employment	740 (11.0)	3716 (10.6)	1.00 (ref)	0.0606	N/A¶	
Not in-paid employment	50 474 (88.3)	26 927 (88.8)	0.96 (0.91 to 1.01)		N/A¶	
Missing	391 (0.7)	202 (0.6)			N/A¶	
Place of residence						
Urban	9455 (18.4)	7127 (27.6)	1.00 (ref)	<0.001	1.00 (ref)	<0.001
Rural	48 811 (81.6)	23 718 (72.4)	1.69 (1.62 to 1.76)		1.26 (1.21 to 1.32)	
Missing	0 (0)	0 (0)			N/A	
Religion						
Hindu	47 322 (80.3)	25 948 (82.4)	1.00 (ref)	<0.001	1.00 (ref)	<0.001
Muslim	9209 (17.8)	4319 (16.2)	1.13 (1.08 to 1.18)		1.16 (1.12 to 1.22)	
Christian	625 (0.9)	253 (0.7)	1.33 (1.11 to 1.60)		1.39 (1.20 to 1.62)	
Others	1016 (0.9)	277 (0.6)	1.48 (1.23 to 1.76)		2.05 (1.78 to 2.36)	
Missing	94 (0.2)	48 (0.1)	1.23 (0.78 to 1.95)		N/A††	
Social group						
Others	41 708 (72.4)	22 826 (74.8)	1.00 (ref)	<0.001	1.00 (ref)	<0.001
Schedule caste	11 351 (20.5)	5290 (18.9)	1.12 (1.07 to 1.17)		1.08 (1.04 to 1.12)	
Schedule tribe	5113 (7.0)	2681 (6.2)	1.17 (1.10 to 1.25)		0.86 (0.81 to 0.90)	
Missing	94 (0.2)	48 (0.1)	1.24 (0.78 to 1.97)		N/A**	
Wealth/asset index						
5: Highest	11 977 (20.6)	8684 (30,2)	1.00 (ref)	<0.001	1.00 (ref)	<0.001
4	11 496 (19.8)	6726 (21.8)	1.29 (1.23 to 1.36)		1.08 (1.03 to 1.13)	
3	11 012 (19.8)	5421 (17.5)	1.57 (1.49 to 1.66)		1.18 (1.13 to 1.24)	
2	10 113 (16.8)	4521 (13.7)	1.67 (1.58 to 1.76)		1.24 (1.18 to 1.31)	
1: Lowest	9443 (15.7)	3348 (9.8)	2.02 (1.90 to 2.14)		1.45 (1.38 to 1.53)	
Missing	4225 (7.2)	2145 (7.1)	1.46 (1.36 to 1.57)		1.33 (1.25 to 1.42)	
Health seeking behaviour						
Antenatal care use						
No	46 392 (79.7)	22 656 (71.2)	1.00 (ref)	<0.001	1.00 (ref)	<0.001
Yes	11 874 (20.3)	8189 (28.8)	0.63 (0.60 to 0.66)		0.69 (0.67 to 0.72)	
Missing	(0)	(0)	N/A		N/A	
Family characteristics						
Number of surviving children at the point of interview						
0	2203 (3.9)	703 (1.9)	1.85 (1.66 to 2.07)	<0.001	1.30 (1.16 to 1.46)	<0.001
1–3	35 751 (59.8)	20 165 (61.5)	1.00 (ref)		1.00 (ref)	
4 or more	8991 (17.2)	4924 (19.8)	0.94 (0.90 to 0.99)		0.96 (0.91 to 1.00)	
Missing	11 321 (19.2)	5053 (16.8)	1.06 (1.01 to 1.11)		0.79 (0.68 to 0.93)	
Proportion of surviving female children among total surviving children						
0%	12 596 (21.0)	7206 (21.8)	1.00 (ref)	<0.001	1.00 (ref)	<0.001
10%–30%	6148 (11.6)	3554 (12.4)	0.97 (0.91 to 1.03)		0.97 (0.92 to 1.03)	
40%–60%	11 704 (20.4)	6481 (21.2)	0.99 (0.94 to 1.05)		1.05 (1.00 to 1.10)	
70%–90%	4736 (8.3)	2749 (9.0)	0.96 (0.90 to 1.03)		1.00 (0.94 to 1.06)	
100%	10 343 (17.0)	5379 (16.1)	1.10 (1.04 to 1.15)		1.12 (1.07 to 1.17)	
Missing	12 739 (21.6)	5476 (19.6)	1.14 (1.08 to 1.20)		1.43 (1.23 to 1.66)	
Family planning						
Use of family planning methods at the point of interview						
No	24 762 (42.2)	10 325 (34.7)	1.00 (ref)	<0.001	1.00 (ref)	<0.001
Yes	22 823 (39.1)	16 101 (49.4)	0.65 (0.63 to 0.67)		0.69 (0.66 to 0.71)	
Missing	10 681 (18.8)	4419 (15.9)	0.97 (0.92 to 1.02)		1.02 (0.97 to 1.06)	
**Others**						
Self-reported mental illness						
No	57 752 (99.0)	30 589 (99.1)	1.0 (ref)	0.5773	N/A¶	
Yes	420 (0.8)	207 (0.7)	1.07 (0.88 to 1.31)		N/A¶	
Missing	94 (0.2)	49 (0.1)	1.20 (0.76 to 1.90)		N/A¶	

*Frequencies are unweighted (true counts). Rates/proportions are weighted for survey design and clustering effects.

†The 95% CIs were calculated using linearised standard errors p value for a t-test showing the significance level for the overall univariable regression model.

‡Multivariable logistic regression, adjusting for the other variables/potential risk factors in the final model.

§P value for a Wald test indicating whether the incremental adjustment for an independent variable significantly improves the fit of the model.

¶N/As are shown because these variables were not included in the final multivariable logistic model.

**N/As are shown because the OR associated with the missing category was extremely large, which can be explained by the small number of missing values in each group.

††N/A is shown because the OR was omitted due to collinearity between missing categories.

‡‡P value for a t-test showing the significance level for the overall univariable regression model.

Compared with women aged 25–29 years, the adjusted odds of unsafe abortion were 13% higher for younger women (20–24 years), and 18% lower for older women (35–39 and 40–44 years). Women living in rural settings had 26% higher odd of unsafe abortion compared with women living in urban settings. Muslim, Christian, or ‘other’ stated religion were associated with increased odds of unsafe abortion compared with Hindu. Education was inversely associated with unsafe abortion; women with no education were 48% more likely to have an unsafe abortion compared with women with university education or higher. Poorer women (in the lowest asset index quintile) had 45% higher odds of unsafe abortion, compared with women in the highest quintile.

In the univariable analysis, belonging to Schedule caste and Schedule tribe social groups was associated with a higher odds of unsafe abortion compared with the ‘other’ social groups. After adjusting for other risk factors the higher odds of unsafe abortion remained for the Schedule caste group, but for the Schedule tribe social group the association was reversed, with this group having 14% lower odds of unsafe abortion compared with the ‘other’ social group. Further analysis showed that the substantial change in the adjusted OR (aOR) was largely explained by the confounding effect of asset index (or wealth quintiles).

Women who had no children at the point of interview had a 30% higher odds of having unsafe abortion compared with women who had one to three children. Compared with women whose children were all boys, women with all female children had 12% higher odds of having an unsafe abortion. This association was not significant if the woman had at least one surviving male child. On further examination through a subgroup analysis, we did not find any significant association between the number of surviving female children and unsafe abortion in households with all female children.

After adjusting for other risk factors, reported use of family planning methods at the point of survey was associated with a 21% lower odds of unsafe abortion. Women who had used ANC had 31% lower odds of unsafe abortion compared with those who did not use ANC. The results of complete case analysis and multiple imputations were not materially different from the ‘missing indicator’ model (online supplementary table S5).

### Risk factors for abortion-related maternal deaths in India

Of the six potential risk factors investigated (maternal age, place of residence, religion, social group, wealth/asset index, gestational month), five were statistically significantly associated with abortion-related death (table 4). We found a non-linear (U-shaped) association between mother’s age and abortion-related death (online supplementary figure S4). Compared with women aged 25–29 years, the aOR for abortion-related death was approximately eight times higher for women aged <20 years, and two times and four times higher for women aged 40–44, and ≥45 years, respectively. Women belonging to a Schedule tribe social group were four times as likely to die during or after having an abortion compared with the reference ‘other’ social group, but the association was not statistically significant for women belonging to a Schedule caste social group (OR: 1.38; 95% CI 0.52 to 3.66). Living in rural areas was associated with a higher odd of abortion-related death (aOR: 3.28; 95% CI 1.76 to 6.11). While the results of our missing indicator analysis were not materially different from the other models, one notable difference was that in the complete case model women who had an abortion at a gestational age of ≥5 months had a significantly higher odds of dying compared with women who had an abortion before 5 months (aOR: 4.35; 95% CI 2.53 to 7.50) (online supplementary table S6).

**Table 4:** Unadjusted and adjusted associations between sociodemographic characteristics, gestational age at abortion and abortion-related maternal death

	Women who died of abortion* (n=253)	Women who survived abortion* (n=89 194)	Unadjusted OR (95% CI)† Missing indicator (n=89 443)	P value	Adjusted OR ‡ (95% CI)† Missing indicator (n=89 443)	P value§
Maternal age (years)						
15–19	36 (13.5)	3591 (4.0)	6.53 (1.71 to 24.95)	<0.001	7.79 (2.73 to 22.23)	<0.001
20–24	59 (23.6)	25 775 (28.9)	3.13 (1.06 to 9.22)		4.29 (2.00 to 9.20)	
25–29	54 (19.9)	29 310 (32.3)	1.00 (ref)		1.00 (ref)	
30–34	42 (17.0)	17 640 (19.8)	0.8 (0.31 to 2.04)		1.12 (0.63 to 1.96)	
35–39	38 (15.6)	8975 (10.2)	4.1 (1.18 to 14.28)		5.95 (2.11 to 16.81)	
40–44	16 (5.7)	2903 (3.5)	1.51 (0.55 to 4.16)		2.07 (1.06 to 4.07)	
≥45	8 (4.8)	1000 (1.3)	3.52 (1.1 to 11.22)		4.35 (1.77 to 10.67)	
Missing	0 (0.0)	0 (0)	N/A		N/A	
Place of residence						
Urban	24 (13.0)	16 601 (21.4)	1.00 (ref)	<0.001	1.00 (ref)	<0.001
Rural	225 (86.2)	72 593 (78.6)	4.56 (2.46 to 8.47)		3.28 (1.76 to 6.11)	
Missing	4 (0.4)	0 (0)	N/A		N/A	
Religion						
Hindu	202 (81.5)	73 340 (81.0)	1.00 (ref)	0.4193	1.00 (ref)	0.1618
Others	48 (18.9)	15 712 (22.2)	0.82 (0.33 to 2.02)		0.94 (0.35 to 2.55)	
Missing	3 (0.8)	142 (0.2)	N/A		N/A¶	
Social group						
Others	167 (68.5)	64 591 (73.2)	1.00 (ref)	0.0138	1.00 (ref)	0.0035
Schedule caste	48 (18.5)	16 656 (19.9)	1.38 (0.52 to 3.66)		1.25 (0.43 to 3.60)	
Schedule tribe	35 (11.8)	7805 (6.8)	4.77 (1.74 to 13.05)		4.06 (1.39 to 11.87)	
Missing	3 (1.1)	143 (0.2)	N/A		N/A¶	
Wealth/asset index						
5: Highest	45 (22.4)	20 678 (23.3)	1.00 (ref)	<0.001	1.00 (ref)	<0.001
4	47 (16.9)	18 242 (20.4)	0.86 (0.53 to 1.40)		0.65 (0.39 to 1.10)	
3	52 (18.8)	16 452 (19.1)	5.81 (2.76 to 12.22)		3.81 (1.86 to 7.83)	
2	54 (18.7)	14 646 (15.9)	2.18 (0.85 to 5.61)		1.33 (0.50 to 3.57)	
1: Lowest	44 (18.7)	12 799 (14.1)	3.56 (1.42 to 8.92)		2.08 (0.73 to 5.94)	
Missing	11 (4.5)	6377 (7.1)	N/A		0.33 (0.16 to 0.70)	
Gestational month						
<5 months	205 (77.8)	86 792 (97.2)	1.00 (ref)	<0.001	1.00 (ref)	<0.001
≥5 months	21 (10.1)	2337 (2.7)	1.87 (0.97 to 3.61)		1.58 (0.80 to 3.13)	
Missing	27 (6.0)	65 (0.1)	N/A		N/A¶	

*Frequencies are unweighted (true counts). Rates/proportions are weighted for survey design and clustering effects.

†The 95% CIs were calculated using linearised standard errors.

‡Multivariable logistic regression, adjusting for the other variables/potential risk factors in the final model.

§ P value for a Wald test indicating whether the incremental adjustment for an independent variable significantly improves the fit of the model.

¶N/As are shown because the OR associated with the missing category was extremely large, which can be explained by the small number of missing values in each group.

**P value for a t-test showing the significance level for the overall univariable regression model.

Further analysis of factors contributing to abortion-related maternal death showed that a third of the deaths were due to delays in receiving care at the health facility, 19% were due to inadequate care at health facility and 17% were due to a failure to recognise the seriousness of the condition (online supplementary table S7).

## Discussion

### Main findings

To our knowledge, this study is the first large population-based study to examine unsafe abortion and abortion-related morality in India. The overall rate of abortion was estimated to be 4.8%, ranging from 1.6% to 6.5% among the nine states. Overall, 67.1% of abortions were classified to be unsafe, varying widely across the states with the highest being 78.3% and the lowest being 45.1%. The overall rate of abortion-related death was estimated to be 0.3% and did not vary appreciably across the states.

There were no clinically meaningful differences between women who had an abortion and those who had a live birth, but a significantly lower proportion of the women who had an abortion had ANC, and a higher proportion were educated and belonged to higher socioeconomic status. We found a strong association of unsafe abortion with sociodemographic factors (younger maternal age, lower socioeconomic status, Muslim religion, rural residence, illiteracy, schedule caste social group), healthcare service utilisation (ANC), family characteristics (number of surviving children and proportion of surviving female children) and family planning use. We found that factors associated with unsafe abortions were different from those associated with abortion-related mortality. Teenage women (aged 15–19 years) were found to have the highest risk of abortion-related death in addition to rural residence and lower socioeconomic status.

### Strengths and limitations

Use of data from the AHS, the largest health survey in India, allowed us to conduct an adequately powered, robust investigation of a wide range of potential risk factors. Our findings are reasonably generalisable for high burden states, but may not be generalisable to the rest of India. To our knowledge, this study is the first in India to identify risk factors associated with unsafe abortion and abortion-related death at a population level.

The rate of unsafe abortion and abortion-related mortality may be underestimated due to underreporting of abortion and misclassification of abortion-related death. Women are often reluctant to report induced abortion regardless of the legal context of abortion.^12 13^ Similarly, women might have provided inaccurate information on the three criteria used to classify the safety of abortion. Since the cause of maternal mortality was reported by family members without validation, deaths occurring after having an abortion might have been misclassified as death caused by haemorrhage or pregnancy-related deaths. In an effort to minimise the possibility of misclassification between abortion and stillbirth, women who reported having an abortion after 28 weeks were excluded.

As the survey design was cross-sectional, causality cannot be inferred from the study results. We did not have data on the method of abortion, therefore this could not be used as a criterion for classifying the safety of abortion. However, methods used to estimate unsafe abortion rates vary widely across studies,^2 21–24^ and there are discrepancies between how the WHO definition is worded and how it has been practically applied to measure the burden of unsafe abortion.^25^ Because there were some factors identified in the literature as important risk factors for unsafe abortion (including, eg, sexual behaviour, partners’ approval of abortion, reasons for abortion, pregnancy wantedness and exposure to media), for which data from the AHS were not available, there is a risk of residual confounding. Finally, because death is a rare outcome, this study had restricted statistical power to detect significant associations between risk factors and abortion-related death.

### Other evidence and implications

Our estimates of the prevalence of unsafe abortion in these nine Indian states fit with regional estimates from a study in south-central Asia (57.8%; 95% CI 50.3 to 65.9),^2^ but are much higher than in a study conducted in India using data from the 2015 Health Facilities Survey and national abortion medication sales, which concluded that among 15.6 million abortions occurring in 2015, 0.8 million (5%) abortions were unsafe.^8^ This discrepancy is possibly because in this latter study unsafe abortion was defined only as a surgical abortion performed outside of a health facility, without considering who performed the abortion or when the abortion was performed.

Our results suggest a pervading theme of vulnerability for unsafe abortion related to low socioeconomic status and teenage pregnancy. While there was an increased prevalence of abortion among educated women, the risks of unsafe abortion, and of death related to abortion, were higher among uneducated women, consistent with previous literature.^22 26^ Although the prevalence of abortion was higher among women with higher socioeconomic status, women from lower socioeconomic status, and ‘Schedule caste’ social group, were more likely to have an unsafe abortion, and to die from abortion-related causes. This is consistent with evidence showing that disadvantaged minority groups in Brazil are at a higher risk of unsafe abortion.^22^ Our finding that women belonging to ‘Schedule tribe’ groups were less likely to have an unsafe abortion might be explained by different health seeking behaviours in women from these groups or may have arisen due to residual confounding. Nevertheless, the risk of abortion-related death was higher in both social groups, indicating the possibility of disparities in access to adequate healthcare for management of abortion complications.

The importance of access to adequate healthcare is also highlighted by our findings on place of residence. Compared with women in urban settings, women living in rural settings were more likely to have an unsafe abortion and more likely to die from an abortion-related cause. More than half (56.28%) of the abortion-related deaths in this study were due to a lack of access to appropriate healthcare (ie, delay in receiving healthcare at facility, inadequate care at health facility and lack of transport to the facility). About 70% of India’s population live in rural settings, but safe abortion services are rarely available at rural facilities.^21^ In the state of Rajasthan, for example, rural settings had an estimated 0.85 certified abortion facilities per 100 000 population, compared with 3.65 in urban settings.^27^

Lack of access to appropriate health services is also reflected in our results in other ways. Our complete case analysis showed that gestational age at the time of the abortion was found to be one of the strongest risk factors for abortion-related mortality, which is consistent with the finding of one study conducted in the USA.^11^ Although it was not possible to examine the safety of abortion among women who died, this variable serves as a proxy for unsafe abortion, supporting the evidence that an abortion-related death is most likely to occur after an unsafe abortion.^14^ The process of seeking an abortion, or care for complications of spontaneous or induced abortion, can involve multiple visits to different providers, resulting in delays, with potentially devastating consequences.^28–30^ In India, preventing unwanted pregnancies through family planning is a key strategy for reducing abortion rates.^31 32^ Access to family planning services, may also be important for reducing the risks of having an unsafe abortion.^23 33 34^ Finally, our results also suggest that antenatal check-ups may be important in reducing the risk of maternal morbidity and mortality resulting from complications, even if they plan to seek an abortion.

Beside socioeconomic factors, women’s age was significantly associated with unsafe abortion and abortion-related death. Younger women (≤24 years) were at a higher risk of unsafe abortion and risk of abortion-related death was highest among teenage women (15–19 years). Older women (≥30 years) were less likely to have an unsafe abortion, but were more likely to die as a result of an abortion. Other studies, in Bangladesh^26^ and Nigeria,^35^ found similar results in relation to maternal age and unsafe abortion. Although female selective abortion (FSA) is illegal in India, the practice is still prevalent.^36–38^ Our finding that women with no male children were more likely to have an unsafe abortion compared with women who had at least one male child is consistent with FSA being sought from unregistered and unqualified abortion providers.^3 39^

## Conclusion

The high estimated prevalence of unsafe abortion in India demonstrates a critical public health problem. Consistent with research in other low-and-middle income countries (LMICs), our results demonstrate that socioeconomic vulnerability, teenage pregnancy and inadequate access to healthcare services combine to leave large numbers of women at risk of unsafe abortion and abortion-related death. There is an urgent need to ensure adequate access to family planning, early abortion services and adequate care for management of postabortion complications, particularly in disadvantaged areas. Further research providing empirical evidence on the barriers to safe abortion services in India is essential to reduce unsafe abortions and deaths, particularly in populations identified to be at a higher risk.

## References

[1] Grimes DA, Benson J, Singh S, et al Unsafe abortion: the preventable pandemic. The Lancet 2006;368:1908–19. 10.1016/S0140-6736(06)69481-6 17126724

[2] Ganatra B, Gerdts C, Rossier C, et al Global, regional, and subregional classification of abortions by safety, 2010–14: estimates from a Bayesian hierarchical model. The Lancet 2017;390:2372–81. 10.1016/S0140-6736(17)31794-4 PMC5711001 28964589

[3] WHO Unsafe abortion: global and regional estimates of the incidence of unsafe abortion and associated mortality in 2008 Geneva. Available: http://www.who.int/reproductivehealth/publications/unsafe_abortion/9789241501118/en/[Accessed 5 Feb 2019].

[4] Khan KS, Wojdyla D, Say L, et al Who analysis of causes of maternal death: a systematic review. The Lancet 2006;367:1066–74. 10.1016/S0140-6736(06)68397-9 16581405

[5] POPLINE by K4Health Abortion in India: what does the National family health survey tell us?. Available: https://www.popline.org/node/280482 [Accessed 5 Feb 2019].

[6] Haddad LB, Nour NM Unsafe abortion: unnecessary maternal mortality. Rev Obstet Gynecol 2009;2:122–6. 19609407 PMC2709326

[7] POPLINE K4Health Realizing reproductive choice and rights: Abortion and contraception in India. Available: https://www.popline.org/node/562996 [Accessed 5 Feb 2019].

[8] Singh S, Shekhar C, Acharya R, et al The incidence of abortion and unintended pregnancy in India, 2015. The Lancet Global Health 2018;6:e111–20. 10.1016/S2214-109X(17)30453-9 29241602 PMC5953198

[9] Duggal R, Ramachandran V The abortion assessment Project—India: key findings and recommendations. Reproductive Health Matters 2004;12:122–9. 10.1016/S0968-8080(04)24009-5 15938165

[10] Adler AJ, Filippi V, Thomas SL, et al Quantifying the global burden of morbidity due to unsafe abortion: magnitude in hospital-based studies and methodological issues. International Journal of Gynecology & Obstetrics 2012;118(Suppl 2):S65–S77. 10.1016/S0020-7292(12)60003-4 22920625

[11] Bartlett LA, Berg CJ, Shulman HB, et al Risk factors for legal induced Abortion–Related mortality in the United States. Obstetrics & Gynecology 2004;103:729–37. 10.1097/01.AOG.0000116260.81570.60 15051566

[12] Jones EF, Forrest JD Underreporting of abortion in surveys of U.S. women: 1976 to 1988. Demography 1992;29:113–26. 10.2307/2061366 1547898

[13] Udry JR, Gaughan M, Schwingl PJ, et al A medical Record linkage analysis of abortion underreporting. Family Planning Perspectives 1996;28:228–31. 10.2307/2135842 8886766

[14] Rees H, Katzenellenbogen J, Shabodien R, et al The epidemiology of incomplete abortion in South Africa. South African Medical Journal 1997;87:432–7. 9254785

[15] Altijani N, Carson C, Choudhury SS, et al Stillbirth among women in nine states in India: rate and risk factors in study of 886,505 women from the annual health survey. BMJ Open 2018;8:e022583 10.1136/bmjopen-2018-022583 PMC6231551 30413502

[16] Office of Registrar General & Census Commissioner MoHA GoI Annual health survey report: a report on core and vital health indicators Part I, 2014.

[17] Vyas S, Kumaranayake L Constructing socio-economic status indices: how to use principal components analysis. Health Policy and Planning 2006;21:459–68. 10.1093/heapol/czl029 17030551

[18] Filmer D, Pritchett LH Estimating wealth effects without expenditure Data-or tears: an application to educational Enrollments in states of India. Demography 2001;38:115–32. 11227840 10.1353/dem.2001.0003

[19] Wagstaff A, Watanabe N What difference does the choice of Ses make in health inequality measurement? Health Econ. 2003;12:885–90. 10.1002/hec.805 14508873

[20] Pedersen A, Mikkelsen E, Cronin-Fenton D, et al Missing data and multiple imputation in clinical epidemiological research. Clin Epidemiol 2017;9:157–66. 10.2147/CLEP.S129785 28352203 PMC5358992

[21] Banerjee SK, Andersen K Exploring the pathways of unsafe abortion in Madhya Pradesh, India. Global Public Health 2012;7:882–96. 10.1080/17441692.2012.702777 22888792

[22] Fusco CLB, Silva RdeSe, Andreoni S Unsafe abortion: social determinants and health inequities in a vulnerable population in São Paulo, Brazil. Cad. Saúde Pública 2012;28:709–19. 10.1590/S0102-311X2012000400010 22488316

[23] Arambepola C, Rajapaksa LC Risk of unsafe abortion associated with long-term contraception behaviour: a case control study from Sri Lanka. BMC Pregnancy Childbirth 2017;17 10.1186/s12884-017-1376-7 PMC5493010 28662700

[24] Biney AAE, Atiglo DY Examining the association between motivations for induced abortion and method safety among women in Ghana. Women & Health 2017;57:1044–60. 10.1080/03630242.2016.1235076 27636891

[25] WHO From concept to measurement: operationalizing WHO’s definition of unsafe abortion. Available: http://www.who.int/bulletin/volumes/92/3/14-136333/en/ [Accessed 5 Feb 2019]. 10.2471/BLT.14.136333 PMC3949603 24700971

[26] DaVanzo J, Rahman M Pregnancy termination in Matlab, Bangladesh: trends and correlates of use of safer and less-safe methods. IPSRH 2014;40:119–26. 10.1363/4011914 25271647

[27] Iyengar K, Iyengar SD Improving access to safe abortion in a rural primary care setting in India: experience of A service delivery intervention. Reprod Health 2016;13. 10.1186/s12978-016-0157-5 PMC4863363 27165519

[28] Elul B, Bracken H, Verma S, et al Unwanted pregnancy and induced abortion in Rajasthan, India: a qualitative exploration 2004.

[29] Ganatra B, Manning V, Pallipamulla SP Availability of medical abortion pills and the role of chemists: a study from Bihar and Jharkhand, India. Reprod Health Matters 2005;13:65–74. 10.1016/S0968-8080(05)26215-8 16291487

[30] Zavier AJF, Jejeebhoy S, Kalyanwala S Factors associated with second trimester abortion in rural Maharashtra and Rajasthan, India. Global Public Health 2012;7:897–908. 10.1080/17441692.2011.651734 22263668

[31] Marston C, Cleland J Relationships between contraception and abortion: a review of the evidence. International Family Planning Perspectives 2003;29:6–13. 10.2307/3180995 12709307

[32] Miller G, Valente C Population policy: Abortion and modern contraception are substitutes. Demography 2016;53:979–1009. 10.1007/s13524-016-0492-8 27383846 PMC5016566

[33] Maina BW, Mutua MM, Sidze EM Factors associated with repeat induced abortion in Kenya. BMC Public Health 2015;15 10.1186/s12889-015-2400-3 PMC4604103 26459344

[34] WHO The effects of contraception on obstetric outcomes. Available: http://www.who.int/reproductivehealth/publications/family_planning/9241592257/en/ [Accessed 5 Feb 2019].

[35] Bankole A, Sedgh G, Oye-Adeniran BA, et al ABORTION-SEEKING behaviour among Nigerian women. J Biosoc Sci 2008;40:247–68. 10.1017/S0021932007002283 17711597

[36] Hirve SS, law A Abortion law, policy and services in India: a critical review. Reproductive Health Matters 2004;12:114–21. 10.1016/S0968-8080(04)24017-4 15938164

[37] Robitaille M-C, Chatterjee I Sex-selective Abortions and Infant Mortality in India: The Role of Parents’ Stated Son Preference. The Journal of Development Studies 2018;54:47–56. 10.1080/00220388.2016.1241389

[38] Unnithan-Kumar M Female selective abortion – beyond ‘culture’: family making and gender inequality in a globalising India. Culture, Health & Sexuality 2010;12:153–66. 10.1080/13691050902825290 19437177

[39] World Health Organization DoRHaR Preventing gender-biased sex selection: an Interagency statement OHCHR, UNFPA, UNICEF, un women and who, 2011.

