# Supplementary material for: Unsafe abortion and abortion-related death among 1.8 million women in India
Source: BMJ Glob Health. 2019 May 2;4(3):e001491. doi: 10.1136/bmjgh-2019-001491 (PMC6509605; doi:10.1136/bmjgh-2019-001491)
Supplement: Supplementary data [file bmjgh-2019-001491supp001.pdf]

Table S1: Unsafe abortion according to where abortion took place, who performed abortion, and gestation at abortion

	Breakup of unsafe abortion according to three criteria	n	%
**Overall**	Non-health facility	55,148	94.3 (94.1-94.6)
	Non-SBA	42,236	73.3 (72.8-73.7)
	≥ 5 months	2,337	2.7 (2.6-2.8)

SBA – Skilled birth attendant.

Table S2: Description of variables included in the analysis and the order in which variables were added in the multivariable model

Risk factor analysis (Unsafe abortion, abortion-related death or both)	Independent variables	Variable description
Unsafe abortion	Family planning	• Binary: Currently using any type of family planning methods at the time of interview; Not using any type of family planning methods
Both	Place of residence	• Binary: Rural; urban
Both	Social group	• Categorical with 3 levels: Schedule tribe (most disadvantaged); Schedule caste; Others (least disadvantaged)<br>• Schedule tribe and Schedule caste are officially designated groups of historically disadvantaged populations in India<br>• Others refer to the general social class
Both	Religion	• Categorical with 4 levels: Hindu; Muslim (minority); Christian (minority); Others (Sikh, Buddhists etc.)<br>• For abortion-related death analysis, religious minorities (Muslim, Christian, and others) were combined to form one category due to a small number of observations for each group.
Both	Asset index/ wealth	• Quintiles: 5 (richest); 4; 3; 2; 1(poorest)<br>• A measurement of household wealth was constructed from the survey data on household assets using Principle Component Analysis (PCA) and is thought to be a good proxy of economic status.
Unsafe abortion	Number of total surviving children	• Categorical with 3 levels: 0; 1-3; 4 or more
Unsafe abortion	Proportion of female children	• Categorical with 5 levels: 0%; 10-30%; 40-60%; 70-90%; 100%<br>• Proportion of surviving female children was calculated using the number of surviving children and female children.
Both	Maternal age (years)	• Categorical with 7 levels: 15-19; 20-24; 25-29; 30-34; 35-39; 40-44; ≥ 45
Unsafe abortion	Maternal education status	• Categorical with 4 levels: Tertiary and above; Secondary school; Primary school/ below; Illiterate

Unsafe abortion	Antenatal care use	• Binary: Used antenatal care; Did not use antenatal care
Unsafe abortion	Marital status	• Binary: Single; Married
Unsafe abortion	Maternal employment	• Binary: In-paid employment; Not in-paid employment
Abortion-related death	Gestational month of abortion	• Binary: < 5 months (20 weeks); ≥ 5 months (20 weeks)

Table S3: Number and rate of unsafe abortion in nine states in India, using the AHS 2012-2013*

	Women who survived beyond 42 days after the abortion and had sufficient information to examine safety of abortion	Women who had an unsafe abortion	Unsafe abortion rate (95%CI) **
**Overall**	89,111	58,266	67.1 (66.7 to 67.5)
Assam	12,251	5,123	45.1 (44.0 to 46.2)
Bihar	14,491	10,808	73.6 (72.7 to 74.4)
Chhattisgarh	1,641	1,284	78.3 (76.1 to 80.4)
Jharkhand	6,173	4,630	73.9 (72.6 to 75.2)
Madhya Pradesh	7,007	4,419	63.7 (62.5 to 64.9)
Odisha	6,966	4,686	68.6 (67.4 to 69.8)
Rajasthan	4,878	2,699	55.8 (52.1 to 55.4)
Uttarakhand	6,464	4,132	62.2 (60.4 to 63.9)
Uttar Pradesh	29,240	20,485	69.3 (68.8 to 69.9)

* Frequencies are unweighted (true counts). Rates/proportions are weighted for survey design and clustering effects.
** The unsafe abortion rate is the number of unsafe abortion per 100 abortions

Table S4: Number and rate of abortion-related death by nine states in India, using the AHS 2012-2013*

	Women who had an abortion, including those who died after having an abortion	Women who died during/after having an abortion	Rate of abortion-related death, % (95%CI)
**Overall**	89,194	253	0.3 (0.2 to 0.3)
Assam	12,264	42	0.5 (0.3 to 0.7)
Bihar	14,496	66	0.4 (0.3 to 0.5)
Chhattisgarh	1,641	17	1.1 (0.6 to 1.8)
Jharkhand	6,194	18	0.2 (0.1 to 0.4)
Madhya Pradesh	7,014	27	0.4 (0.3 to 0.6)
Odisha	6,977	7	0.1 (0.1 to 0.3)

Rajasthan	4,889	8	0.1 (0.1 to 0.3)
Uttarakhand	6,464	6	0.1 (0.0 to 0.4)
Uttar Pradesh	29,255	62	0.2 (0.1 to 0.3)
* Frequencies are unweighted (true counts). Rates/proportions are weighted for survey design and clustering effects.<br>** The rate of abortion-related death is the number of abortion-related death per 100 abortions			

Table S5: Sensitivity analysis for unsafe abortion: risk factor analysis for unsafe abortion

	**Adjusted OR (95%CI)**<br>**Missing indicator**<br>(n=89,111)	**Adjusted OR (95%CI)**<br>**Complete case analysis**<br>(n=56,093)	**Adjusted OR (95%CI)**<br>**Multiple imputation**<br>(n=88,967)
**Maternal age (years)**			
15-19	1.09 (1.00 to 1.18)	1.17 (0.97 to 1.41)	1.14 (0.97 to 1.33)
20-24	1.13 (1.09 to 1.18)	1.18 (1.11 to 1.26)	1.19 (1.13 to 1.25)
25-29	1.00 (ref)	1.00 (ref)	1.00 (ref)
30-34	0.88 (0.84 to 0.92)	0.88 (0.83 to 0.93)	0.88 (0.84 to 0.92)
35-39	0.82 (0.78 to 0.87)	0.87 (0.81 to 0.94)	0.84 (0.79 to 0.89)
40-44	0.82 (0.75 to 0.89)	0.90 (0.79 to 1.01)	0.82 (0.75 to 0.90)
≥ 45	0.95 (0.83 to 1.09)	0.93 (0.76 to 1.12)	1.00 (0.85 to 1.18)
Missing	N/A	N/A	N/A
**Marital status**			
Married	N/A	N/A	N/A
Single	N/A	N/A	N/A
Missing	N/A	N/A	N/A
**Maternal Education**			
Tertiary and above	1.00 (ref)	1.00 (ref)	1.00 (ref)
Secondary school	0.94 (0.88 to 1.00)	0.90 (0.81 to 1.00)	0.85 (0.78 to 0.92)
Primary school / below	1.13 (1.05 to 1.20)	1.04 (0.93 to 1.17)	1.04 (0.95 to 1.14)
Illiterate	1.48 (1.39 to 1.59)	1.32 (1.17 to 1.48)	1.41 (1.29 to 1.55)
Missing	NA *	NA	N/A
**Maternal employment status**			
In-paid employment	N/A	N/A	N/A
Not in-paid employment	N/A	N/A	N/A
Missing	N/A	N/A	N/A
**Place of residence**			
Urban	1.00 (ref)	1.00 (ref)	1.00 (ref)
Rural	1.26 (1.21 to 1.32)	1.35 (1.27 to 1.43)	1.19 (1.14 to 1.25)
Missing	N/A	N/A	
**Religion**			
Hindu	1.00 (ref)	1.00 (ref)	1.00 (ref)
Muslim	1.16 (1.12 to 1.22)	1.20 (1.13 to 1.28)	1.20 (1.14 to 1.27)
Christian	1.39 (1.20 to 1.62)	1.12 (0.89 to 1.40)	1.24 (1.03 to 1.50)
Others	2.05 (1.78 to 2.36)	1.22 (0.98 to 1.52)	1.91 (1.61 to 2.27)
Missing	N/A **	N/A **	

**Social group**			
Others	1.00 (ref)	1.00 (ref)	1.00 (ref)
Schedule caste	1.08 (1.04 to 1.12)	1.04 (0.98 to 1.11)	1.08 (1.03 to 1.13)
Schedule tribe	0.86 (0.81 to 0.90)	0.91 (0.84 to 0.99)	0.78 (0.74 to 0.84)
Missing	NA*	NA	N/A
**Wealth/ Asset index**			
5: Highest	1.00 (ref)	1.00 (ref)	1.00 (ref)
4	1.08 (1.03 to 1.13)	1.10 (1.03 to 1.18)	1.05 (1.00 to 1.11)
3	1.18 (1.13 to 1.24)	1.20 (1.12 to 1.29)	1.12 (1.06 to 1.18)
2	1.24 (1.18 to 1.31)	1.26(1.17 to 1.35)	1.18 (1.11 to 1.26)
1: Lowest	1.45 (1.38 to 1.53)	1.45 (1.34 to 1.57)	1.40 (1.31 to 1.50)
Missing	1.33 (1.25 to 1.42)	NA	
**Antenatal care use**			
No	1.00 (ref)	1.00 (ref)	1.00 (ref)
Yes	0.69 (0.67 to 0.72)	0.63 (0.60 to 0.66)	0.73 (0.70 to 0.76)
Missing	N/A	N/A	N/A
**Self-reported mental illness**			
No	N/A	N/A	N/A
Yes	N/A	N/A	N/A
Missing	N/A	N/A	N/A
**Number of surviving children at the point of interview**			
0	1.30 (1.16 to 1.46)	1.33 (1.10 to 1.61)	1.43 (1.23 to 1.67)
1-3	1.00 (ref)	1.00 (ref)	1.00 (ref)
4 or more	0.96 (0.91 to 1.00)	0.88 (0.83 to 0.94)	0.96 (0.91 to 1.01)
Missing	0.79 (0.68 to 0.93)	N/A	N/A
**Proportion of surviving female children among total surviving children**			
0%	1.00 (ref)	1.00 (ref)	1.00 (ref)
10-30%	0.97 (0.92 to 1.03)	1.03 (0.96 to 1.14)	1.02 (0.96 to 1.09)
40-60%	1.05 (1.00 to 1.10)	1.08 (1.01 to 1.15)	1.08 (1.03 to 1.14)
70-90%	1.00 (0.94 to 1.06)	1.04 (0.95 to 1.13)	1.03 (0.96 to 1.10)
100%	1.12 (1.07 to 1.17)	1.13 (1.05 to 1.20)	1.12 (1.06 to 1.18)
Missing	1.43 (1.23 to 1.66)	N/A	N/A
**Family planning (currently at the point of interview)**			
No	1.00 (ref)	1.00 (ref)	1.00 (ref)
Yes	0.69 (0.66 to 0.71)	0.70 (0.67 to 0.73)	0.65 (0.63 to 0.67)
Missing	1.02 (0.97 to 1.06)	N/A	N/A

* N/As are shown because the OR associated with the missing category was extremely large, which can be explained by the small number of missing values in each group.
** Omitted due to collinearity

Table S6: Sensitivity analysis for abortion-related death: risk factor analysis for abortion-related death

	**Adjusted OR (95%CI)** **Complete case analysis** (n=82,961)	**Adjusted OR (95%CI)** **Missing indicator** (n=89,443)	**Adjusted OR (95%CI)** **Multiple imputation †**
**Maternal age (years)**			
15-19	5.01 (2.91 to 8.62)	7.79 (2.73 to 22.23)	N/A
20-24	1.14 (0.71 to 1.84)	4.29 (2.00 to 9.20)	N/A
25-29	1.00 (ref)	1.00 (ref)	N/A
30-34	1.36 (0.80 to 2.32)	1.12 (0.63 to 1.96)	N/A
35-39	2.28 (1.34 to 3.90)	5.95 (2.11 to 16.81)	N/A
40-44	2.13 (1.07 to 4.24)	2.07 (1.06 to 4.07)	N/A
≥ 45	4.99 (1.92 to 12.96)	4.35 (1.77 to 10.67)	N/A
Missing	N/A	N/A	N/A
**Place of residence**			
Urban	1.00 (ref)	1.00 (ref)	N/A
Rural	1.57 (0.91 to 2.69)	3.28 (1.76 to 6.11)	N/A
Missing	N/A	N/A	N/A
**Religion**			
Hindu	1.00 (ref)	1.00 (ref)	N/A
Others	1.22 (0.81 to 1.84)	0.94 (0.35 to 2.55)	N/A
Missing	N/A	N/A **	N/A
**Social group**			
Others	1.00 (ref)	1.00 (ref)	N/A
Schedule caste	1.05 (0.68 to 1.63)	1.25 (0.43 to 3.60)	N/A
Schedule tribe	1.85 (1.16 to 2.97)	4.06 (1.39 to 11.87)	N/A
Missing	N/A	N/A *	N/A
**Wealth/ Asset index**			
5: Highest	1.00 (ref)	1.00 (ref)	N/A
4	0.75 (0.45 to 1.25)	0.65 (0.39 to 1.10)	N/A
3	0.86 (0.52 to 1.42)	3.81 (1.86 to 7.83)	N/A
2	0.94 (0.57 to 1.56)	1.33 (0.50 to 3.57)	N/A
1: Lowest	0.94 (0.55 to 1.61)	2.08 (0.73 to 5.94)	N/A
Missing	N/A	0.33 (0.16 to 0.70)	N/A
**Gestational month**			
< 5 month	1.00 (ref)	1.00 (ref)	N/A
≥ 5 month	4.35 (2.53 to 7.50)	1.58 (0.80 to 3.13)	N/A
Missing	N/A	N/A *	N/A

* N/As are shown because the OR associated with the missing category was extremely large, which can be explained by the small number of missing values in each group.
** Omitted due to collinearity
† N/As are shown because the imputation model did not converge due to the small number of missing values.

Table S7: Number and proportion of factors contributing to abortion-related death

**Factors contributing to death**	**n** (n=253)	**% (95%CI)**
Delay in receiving health care at facility	79	30.33 (23.81 to 37.76)
Inadequate care at health facility	47	19.17 (13.96 to 25.75)
Seriousness of the condition not realised	49	17.63 (12.70 to 23.94)
Lack of funds	28	13.11 (8.78 to 19.14)
Lack of transport to facility	19	6.78 (4.10 to 11.01)
Seriousness of condition realised but no decision made by family members	14	6.48 (3.64 to 11.28)
Others	17	6.50 (3.69 to 11.21)

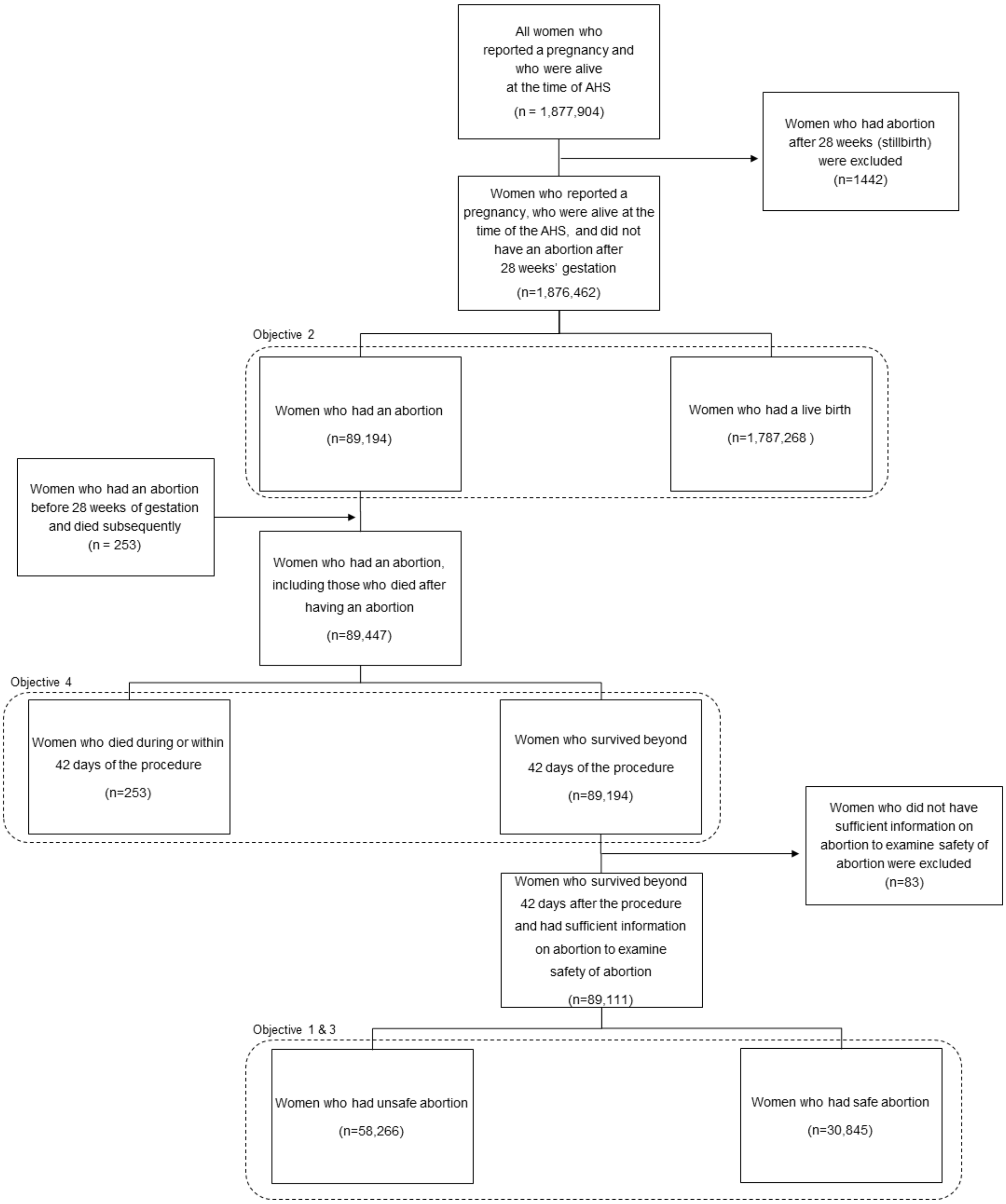

Figure S1: Flow-chart showing the derivation of study sample

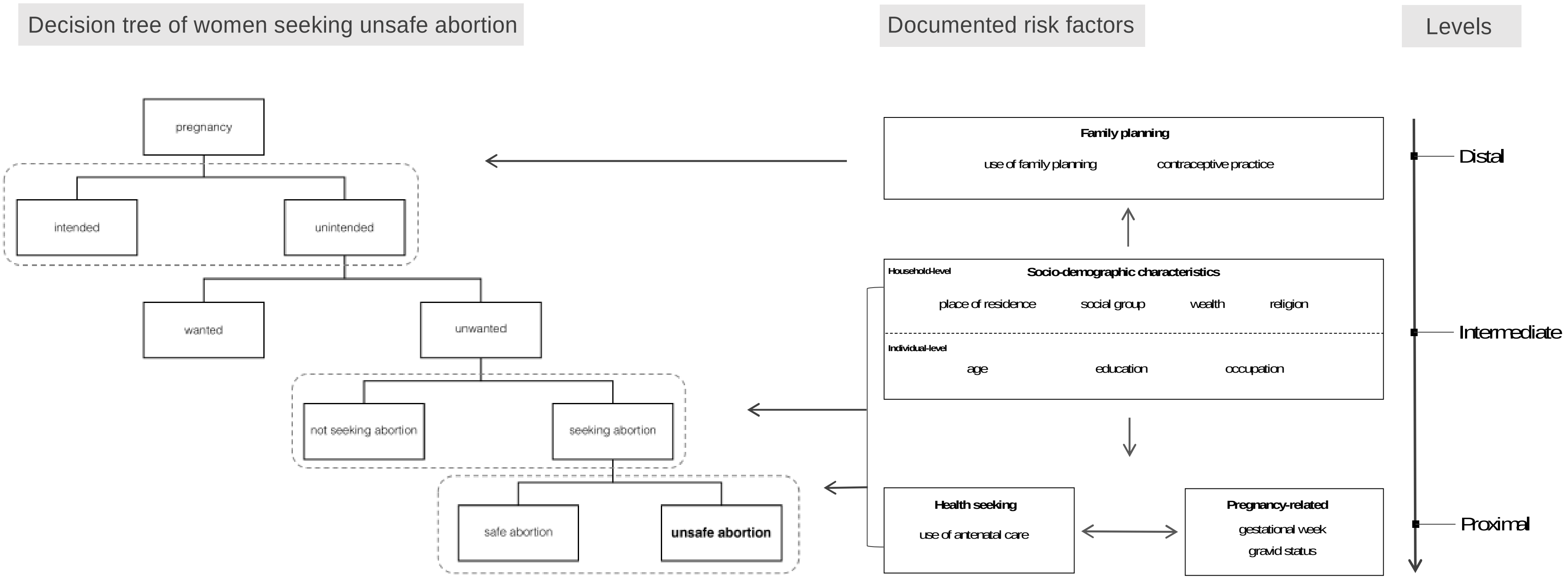

Figure S2: Conceptual framework – risk factors for unsafe abortion

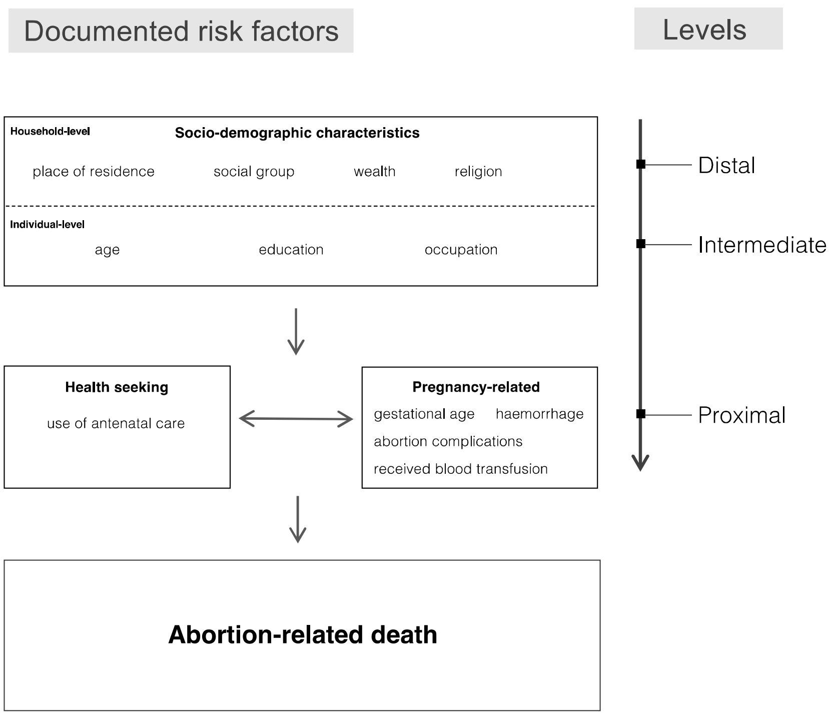

Figure S3: Conceptual framework – risk factors for abortion-related maternal death

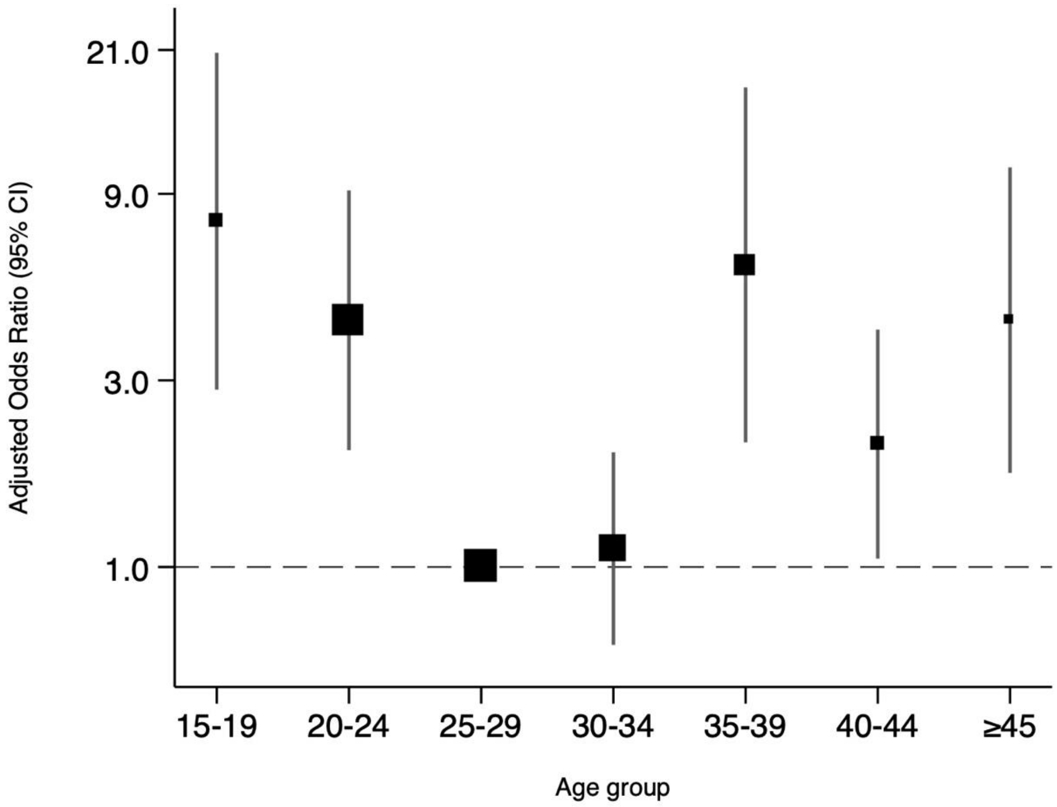

Figure S4: Association of maternal age with abortion-related death
